# Antibody longevity and waning following COVID-19 vaccination in a 1-year longitudinal cohort in Bangladesh

**DOI:** 10.1038/s41598-024-61922-6

**Published:** 2024-05-20

**Authors:** Md. Ahsanul Haq, Anjan Kumar Roy, Razu Ahmed, Rakib Ullah Kuddusi, Monika Sinha, Md. Shamim Hossain, Maya Vandenent, Mohammad Zahirul Islam, Rashid U. Zaman, Md. Golam Kibria, Abdur Razzaque, Rubhana Raqib, Protim Sarker

**Affiliations:** 1 Immunobiology, Nutrition and Toxicology Laboratory, Nutrition Research Division, International Center for Diarrhoeal Disease Research, Bangladesh (icddr, b), Dhaka, 1212 Bangladesh; 2 UNICEF, Dhaka, 1207 Bangladesh; 3 Embassy of Sweden in Bangladesh, Dhaka, 1212 Bangladesh; 4 British High Commission, Dhaka, 1212 Bangladesh; 5 Sheikh Russel Gastroliver Institute and Hospital, Dhaka, 1212 Bangladesh

**Keywords:** Biochemistry, Immunology, Molecular biology, Diseases, Health care

## Abstract

COVID-19 vaccines have been effective in preventing severe illness, hospitalization and death, however, the effectiveness diminishes with time. Here, we evaluated the longevity of antibodies generated by COIVD-19 vaccines and the risk of (re)infection in Bangladeshi population. Adults receiving two doses of AstraZeneca, Pfizer, Moderna or Sinopharm vaccines were enrolled at 2–4 weeks after second dosing and followed-up at 4-monthly interval for 1 year. Data on COVID-like symptoms, confirmed COVID-19 infection, co-morbidities, and receipt of booster dose were collected; blood was collected for measuring spike (S)- and nucleocapsid (N)-specific antibodies. S-specific antibody titers reduced by ~ 50% at 1st follow-up visit and continued to decline unless re-stimulated by booster vaccine dose or (re)infection. Individuals infected between follow-up visits showed significantly lower S-antibody titers at preceding visits compared to the uninfected individuals. Pre-enrolment infection between primary vaccination dosing exhibited 60% and 50% protection against reinfection at 5 and 9 months, respectively. mRNA vaccines provided highest odds of protection from (re)infection up to 5 months (Odds Ratio (OR) = 0.08), however, protection persisted for 9 months in AstraZeneca vaccine recipients (OR = 0.06). In conclusion, vaccine-mediated protection from (re)infection is partially linked to elevated levels of S-specific antibodies. AstraZeneca vaccine provided the longest protection.

## Introduction

The corona virus disease 2019 (COVID-19) pandemic caused by Severe Acute respiratory Coronavirus-2 (SARS-CoV-2) had a catastrophic effect, claiming millions of lives and badly affecting the health systems and economy across the globe. Several COVID-19 vaccines have been developed to fight against the deadly virus. The vaccines have been effective in preventing infections and importantly reducing disease severity, hospitalization and death^1^. On 05 May 2023, the World health Organization (WHO) declared end to COVID-19 as a global health emergency, and at that time about 5.1 billion people worldwide were fully vaccinated^2^. However, people are still getting infected with SARS-CoV-2 even after receiving multiple doses of vaccines^3,4^, raising questions about the longevity of the protective immunity.

Many studies have shown that the effectiveness of the COVID-19 vaccines or immune responses generated by vaccination wanes with time^5,6^, unlike some other vaccines that give lifelong protection such as smallpox or measles vaccines. The ZOE COVID study reported waning of effectiveness of mRNA (Pfizer-BioNTech BNT162b2 and Moderna mRNA 1273) and vector-based (Oxford-AstraZaneca COVID-19) vaccines at around 5 months after administration of two primary doses in community settings^7^. The protection provided by Pfizer vaccine was 91.6% after one month, decreasing to 82% after 5 months; effectiveness of Moderna reduced from 94% to 84% and that of AstraZeneca vaccine declined from 83% to 75.7% at 5 months. Another study performed in 30 million peoples showed increased risk of severe COVID-19 outcome 10 weeks after a second shot of the Pfizer and AstraZeneca vaccine^8^. Because of the waning of binding and neutralizing antibody levels, booster doses were introduced to bolster immune defense against SARS-CoV-2 infections^9,10^. It is currently unknown what levels of antibody responses are being generated against the COVID-19 vaccines and the duration of these antibodies in the peripheral circulation of the community peoples in Bangladesh. For an effective long-term vaccination strategy, it is necessary to comprehend the rate of decay of antibody responses generated by different vaccine types in the community.

In this study, we aimed to assess the SARS-CoV-2 spike(S)-specific antibody response generated by two primary doses of COVID-19 vaccines namely AstraZeneca (Oxford–AstraZeneca COVID‑19 vaccine or Covishield, viral vector-based vaccine), Moderna, Pfizer (mRNA vaccines) and Sinopharm (BBIBP-CorV, inactivated whole virus vaccine), their persistence and the risk of (re)infection in a longitudinal follow-up study in Bangladeshi adult population. Moreover, the dynamics of antibody responses after breakthrough infection and/or after receiving 3rd dose of vaccine have also been evaluated.

## Results

### Characteristics of study participants

A total of 452 participants were enrolled in the study. The baseline demographics of study participants are given in Table 1. The mean age (± standard deviation) of the study participants was 38.1 ± 12.8 years. The average age of the participants of the AstraZeneca group (50.2 ± 12.4 years) was higher than the participants of Pfizer (31.8 ± 8.13 years), Moderna (36.1 ± 10.9 years), and Sinopharm (32.2 ± 8.21 years) groups. In all groups, the male to female ratio was about 2:1 except for the Pfizer group, which had a higher proportion of males (3:1) (Table 1). Majority of the participants were engaged in some sort of service, many were businesspersons or homemakers or students. About half (47%) of the participants had graduate degree.

**Table 1:** Demographic characteristics of the study participants.

	Overall	Pfizer N = 110	Moderna N = 89	AstraZeneca N = 131	Sinopharm N = 122
Age	38.1 ± 12.8	31.8 ± 8.13	36.1 ± 10.9	50.2 ± 12.4	32.2 ± 8.21
Sex					
Male	314(69.5%)	85(77.3%)	60(67.4%)	85(64.9%)	84(68.9%)
Female	138(30.5%)	25(22.7%)	29(32.6%)	46(35.1%)	38(31.2%)
Occupation					
Service	271(60.0%)	65(59.1%)	60(67.4%)	83(63.4%)	63(51.6%)
Business	61(13.5%)	19(17.3%)	8(8.99%)	16(12.2%)	18(14.8%)
Housewife	71(15.7%)	13(11.8%)	13(14.6%)	21(16.0%)	24(19.7%)
Unemployed	8(1.80%)	1(0.91%)	2(2.25%)	2(1.53%)	3(2.46%)
Student	24(5.3%)	9(8.18%)	5(5.62%)	0	10(8.20%)
Others	17(3.80%)	3(2.73%)	1(1.15%)	9(6.87%)	4(3.28%)
Education					
0–5 years	20(4.40%)	9(8.18%)	1(1.12%)	3(2.29%)	7(5.74%)
6–10 years	97(21.5%)	28(25.5%)	14(15.7%)	13(9.92%)	42(34.4%)
11–12 years	38(8.40%)	9(8.18%)	6(6.74%)	7(5.34%)	16(13.1%)
Graduate	107(23.7%)	35(31.8%)	18(20.2%)	29(22.1%)	25(20.5%)
Post-graduate	190(42.0%)	29(26.4%)	50(56.2%)	79(60.3%)	32(26.2%)
Income					
< 30,000 BDT	116(25.7%)	31(28.2%)	15(16.9%)	11(8.40%)	59(48.4%)
31,000–60,000 BDT	154(34.1%)	54(49.1%)	33(37.1%)	26(19.9%)	41(33.6%)
61,000–100,000 BDT	70(15.5%)	12(10.9%)	27(30.3%)	24(18.3%)	7(5.74%)
> 100,000 BDT	112(24.8%)	13(11.8%)	14(15.7%)	70(53.4%)	15(12.3%)
BMI category					
Underweight	16(3.50%)	4(3.64%)	3(3.37%)	2(1.53%)	7(5.74%)
Normal	249(55.1%)	62(56.4%)	47(52.8%)	69(52.7%)	71(58.2%)
Overweight	133(29.4%)	27(24.6%)	32(36.0%)	41(31.3%)	33(27.1%)
Obese	54(11.9%)	17(15.5%)	7(7.87%)	19(14.5%)	11(9.02%)
Comorbidities					
H/O diabetes	46(10.2%)	5(4.55%)	5(5.62%)	31(23.7%)	5(4.10%)
H/O hypertension	54(12.0%)	4(3.64%)	11(12.4%)	35(26.7%)	4(3.28%)

Data are presented as mean ± SD for continuous variables or number with percentage for categorical variables. SD: Standard deviation.

Among all vaccine recipients, 34% belonged to the 60,000–100,000 BDT (Bangladeshi Taka) monthly household income group followed by 26% in < 30,000 BDT and 25% in > 100,000 BDT group. Approximately half of the participants in the Sinopharm group (49%) had monthly income in the lowest quartile (< BDT 30,000), while 53% of the AstraZeneca group belonged to the highest quartile of income (> BDT100,000). More than half of the participants in each vaccine group belonged to normal BMI category, while close to 30% were overweight, and 12% obese (Table 1).

### SARS-COV-2 infection status

**Figure 1:**
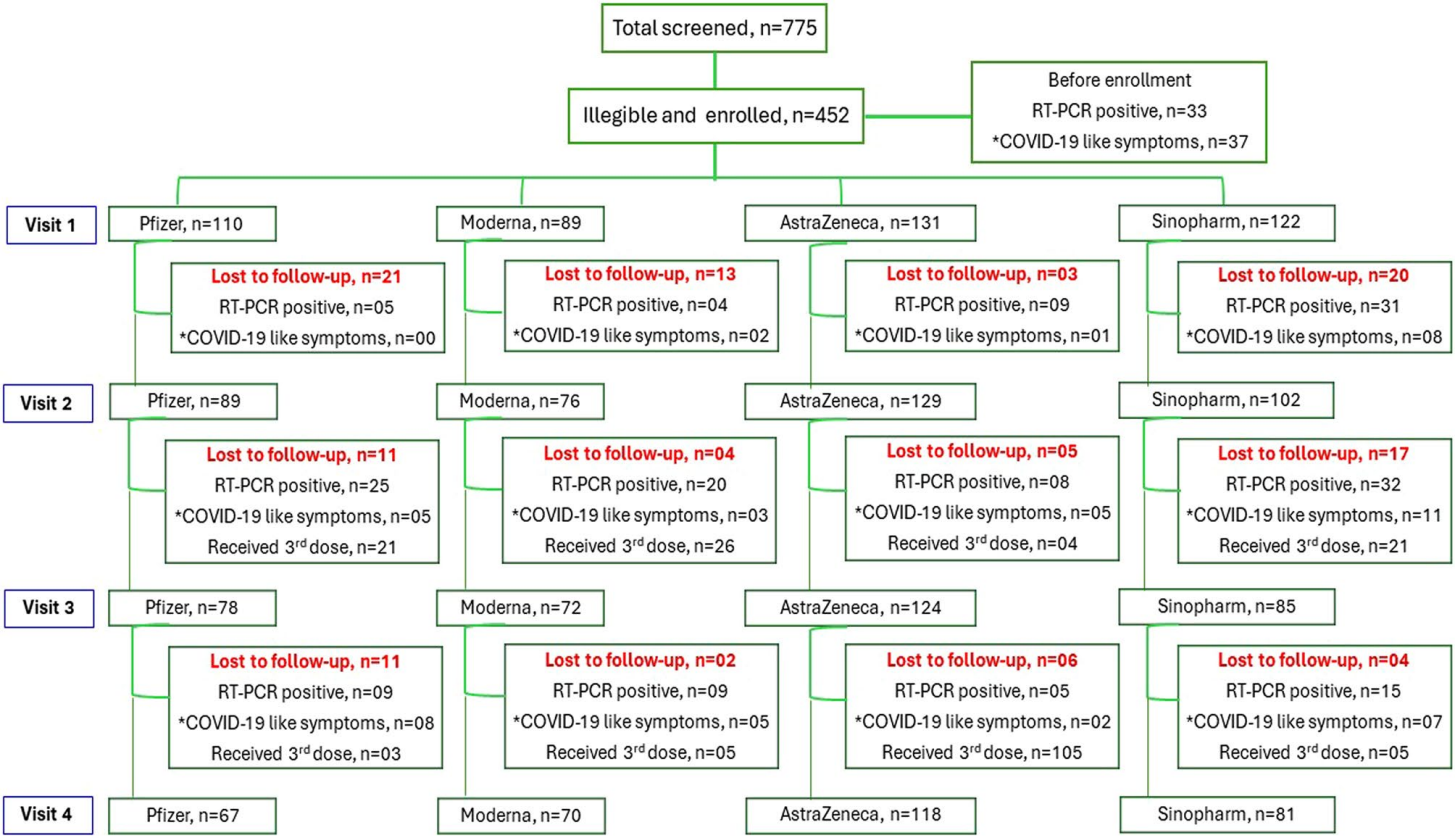
Flow chart showing the enrollment and follow-up of the participants receiving different COVID-19 vaccines as primary doses. Number of participants with RT-PCR-confirmed SARS-CoV-2 infection or COVID-like symptoms before enrollment and during follow-up visits as well as the number of participants, who lost to follow-up or received the third or booster dose are shown. *Participants with COVID-like symptoms were tested positive for SARS-CoV-2 Nucleocapsid (N)-specific antibodies. COVID-19: Corona virus diseases 2019; SARS-CoV-2: Severe Acute respiratory Coronavirus-2; RT-PCR: Reverse transcription polymerase chain reaction.

At enrollment (visit 1), 33 (7.3%) participants across all vaccine groups (12, 6, 4 and 11 in Pfizer, Moderna, AstraZeneca and Sinopharm vaccine groups, respectively) were found to have been infected with SARS-CoV-2 between 1st and 2nd dose of primary vaccination as confirmed by reverse transcription polymerase chain reaction (RT-PCR). Moreover, 37 participants (13, 3, 3 and 18 in Pfizer, Moderna, AstraZeneca and Sinopharm vaccine groups, respectively) had experienced COVID-like symptoms, but they did not undergo RT-PCR testing (Fig. 1). Individuals with COVID-like symptoms were further confirmed of having previous infection with SARS-CoV-2 by the presence of N-antibodies. After enrollment and between each consecutive follow-up visits, i.e. between 1st and 2nd visits, 2nd and 3rd visits, and 3rd and 4th visits, number of SARS-CoV-2 positive participants by RT-PCR were 49, 85 and 38, respectively. Number of participants experiencing COVID-like symptoms, but who did not undergo PCR testing were 11, 24 and 22 between each consecutive study visits, respectively; all of them tested positive for anti-N antibodies. When compared between vaccine groups, Sinopharm vaccine recipients had the highest number of participants with RT-PCR positive breakthrough infection with SARS-CoV-2 between each consecutive study visit (Fig. 1).

### Third dose of vaccination during the study period

Seventy-two participants received the 3rd dose of vaccines between visit 2 and visit 3, while 118 participants received the 3rd dose between visits 3 and 4 (Fig. 1). Among participants who received AstraZeneca vaccine as the primary dose, 58 (53%) received Moderna, 26 (24%) received Pfizer and 25 (23%) received AstraZeneca vaccine as the 3rd dose. Among the group receiving Pfizer vaccine as the primary dose, 15 (65%) received the same vaccine (Pfizer), 6 (26%) received Moderna and 2 (9%) received AstraZeneca as the 3rd dose. Among the group receiving Moderna vaccine as the primary dose, a great majority 24 (77.4%) received the same vaccine, while 4 (13%) received Pfizer, and 3 (10%) received AstraZeneca vaccine as the 3rd dose. Finally, among recipients of Sinopharm as the primary vaccine, 14 (54%) received Moderna, 4 (15.4%) received Pfizer, 6 (23.1%) received AstraZeneca and 2 (7.7%) received Sinopharm vaccine as the 3rd dose.

### Durability of Spike protein (S)- specific antibody titers at different visits post-vaccination

The geometric mean (GM) S-antibody titers at visit 1 were higher in the mRNA vaccine recipients followed by viral vector-based and inactivated vaccine recipients. (Tables 2 and 3). Among group I participants (who remained uninfected with SARS-CoV-2 and did not receive a 3rd dose of vaccine during the study period), there was a significant reduction in S- antibody titers from visit 1 to visit 2 in recipients of each type of vaccines (Table 2). When reduction of S-antibody titers in individual participants were averaged, percentage reduction was found to be 45.7% in Sinopharm, 53% in Pfizer, 56% in Moderna and 57% in AstraZeneca groups. The antibodies further declined at visit 3 (reduced by 35—39% across all vaccine groups compared to visit 2), and continued to decline up to visit 4 (reduced by 26%—45% across all vaccine groups compared to visit 3). It is important to note here that many of the participants either received 3rd dose, or got infected or dropped out from the study, therefore the number of participants in group I were reduced substantially at visits 3 and 4. Only 20 participants completed all follow-up visits without getting re-infected with SARS-COV-2 or receiving 3rd dose of vaccine; an important point to be noted here is that these participants were infected during primary course of vaccination (between 1st and 2nd doses). These participants showed a 64% reduction in mean S-antibody titers after 4 months, 74% after 8 months and 82% after 12 months (from 23,431 ± 19,000 at 1st visit to 8471 ± 7241 at 2nd visit, 6,043 ± 4801 at 3rd visit and 4301 ± 3540 at 4th visit) (Supplementary Fig. 1). This indicated that even with 82% reduction in S-antibody titers from enrollment, these participants remained protected.

**Table 2:** Comparison of S-antibody titers between two consecutive visits in group I participants*.

	Pfizer	Moderna	AstraZeneca	Sinopharm
Between visit 1 & 2				
	n = 84	n = 70	n = 118	n = 63
Visit 1	14,223.3 ± 12.0	17,060.8 ± 20.4	2576.3 ± 31.5	1399.6 ± 13.5
Visit 2	6194.4 ± 22.0	6966.3 ± 12.5	963.8 ± 14.30	707.9 ± 14.20
*P* value	< 0.001	< 0.001	< 0.001	< 0.001
Between visit 2 & 3				
	n = 27	n = 19	n = 99	n = 8
Visit 2	7430.2 ± 13.8	8629.8 ± 16.2	1339.7 ± 12.0	2779.7 ± 20.4
Visit 3	4592.0 ± 15.1	5358.0 ± 14.1	743.0 ± 14.8	1717.9 ± 15.5
*P* value	< 0.001	< 0.001	< 0.001	< 0.001
Between visit 3 & 4				
	n = 10	n = 8		n = 13
Visit 3	6531 ± 16.80	10,280 ± 19.2	–	2153 ± 30.4
Visit 4	3617 ± 20.2	7032 ± 19.7	–	794.0 ± 26.3
*P* value	< 0.001	< 0.001		< 0.001

Data are presented as GM ± SD. Multivariate regression model was used to calculate the estimated mean and the regression model was adjusted by age, sex, household income, occupation, and BMI. *Group I consists of individuals who remained uninfected with SARS-CoV-2 (tested negative by RT-PCR, did not exhibit COVID-like symptoms or negative for N-antibodies) and did not receive the 3rd dose of vaccine during the one-year study period. *BMI*: Body mass index; *SARS-CoV-2*: Severe acute respiratory coronavirus-2; *GM*: Geometric mean; *SD*: Standard deviation; *RT-PCR*: Reverse transcription polymerase chain reaction.

**Table 3:** Comparison of S-antibody titers between two consecutive visits in group II participants^*^.

	Pfizer	Moderna	AstraZeneca	Sinopharm
Between visit 1 & 2				
RT-PCR positive	n = 5	n = 4	n = 9	n = 31
Visit 1	8872 ± 12.2	4193 ± 14.3	1706 ± 8.87	875.2 ± 10.2
Visit 2	11,609 ± 31.5	11,262 ± 13.0	27,996 ± 38.5	4390 ± 12.7
*P* value	< 0.001	0.031	< 0.001	< 0.001
COVID-like symptoms	n = 0	n = 2	n = 1	n = 8
Visit 1	–	9676 ± 11.3	6050	1665 ± 15.3
Visit 2	–	49,522 ± 30.5	8407 ± 23.4	7791 ± 10.5
*P* value		–	–	< 0.001
Between visit 2 & 3				
RT-PCR positive	n = 23	n = 20	n = 8	n = 26
Visit 2	5942 ± 19.2	8270 ± 16.1	327.3 ± 12.2	851.5 ± 11.2
Visit 3	11,695 ± 12.3	23,823 ± 11.0	3548 ± 15.1	7200 ± 12.8
*P* value	< 0.001	< 0.001	< 0.001	< 0.001
COVID-like symptoms	n = 5	n = 3	n = 5	n = 6
Visit 2	5587 ± 11.3	2930 ± 14.1	657.0 ± 9.57	1529 ± 16.3
Visit 3	9068 ± 11.3	10,629 ± 13.1	2828 ± 13.3	3971 ± 12.4
*P* value	< 0.001	0.034	< 0.001	< 0.001
Between visit 3 & 4				
RT-PCR positive	n = 7	n = 6	n = 5	n = 4
Visit 3	4749 ± 21.4	3990 ± 23.5	708.6 ± 14.6	2826 ± 12.7
Visit 4	12,445 ± 19.0	18,355 ± 29.0	14,171 ± 19.3	13,412 ± 16.8
*P* value	< 0.001	< 0.001	< 0.001	< 0.001
COVID-like symptoms	n = 3	n = 5	n = 2	n = 0
Visit 3	5161 ± 23.0	6734 ± 19.4	1945 ± 27.8	–
Visit 4	10,972 ± 21.9	14,662 ± 18.2	19,511 ± 13.0	–
*P* value	0.045	< 0.001	–	

Data are presented as GM ± SD. Multivariate regression model was used to calculate the estimated mean and the regression model was adjusted by age, sex, household income, occupation and BMI. *Group II consists of individuals, who got infected with SARS-CoV-2 (tested positive by RT-PCR or showed COVID-like symptoms and positive for N-antibodies) and did not receive a 3rd dose of vaccine during the study period. *BMI*: Body mass index; *SARS-CoV-2*: Severe acute respiratory coronavirus-2; *RT-PCR*: Reverse transcription polymerase chain reaction; *GM*: Geometric mean; SD: Standard deviation.

Recipients of each type of vaccine among group II participants (who got infected with SARS-CoV-2 and did not receive a 3rd dose of vaccine during the study period), showed lower S-antibody titers compared to group I participants at visit 1. Group II participants showed significantly higher antibody response at visit 2 compared to visit 1 in recipients of each type of vaccine (Table 3). Similarly, participants with SARS-CoV-2 infection or COVID-like symptoms between visits 2 and 3, and between visits 3 and 4 showed significant increases in S-antibody titers in each vaccine group at later of the two consecutive visits.

Multivariate regression analysis showed that vaccinated individuals who became infected (i.e. group II participants) between any two visits (between visits 1 and 2 (Supplementary Fig. 2A); or between visits 2 and 3 (Supplementary Fig. 2B)) had significantly lower S-antibody titers at preceding visits compared to those who remained uninfected (i.e. group I participants). At visits after infection, the S-antibody titers were significantly higher in infected (group II) participants compared to uninfected (group I) participants (Supplementary Fig. 2A & B).

**Figure 2:**
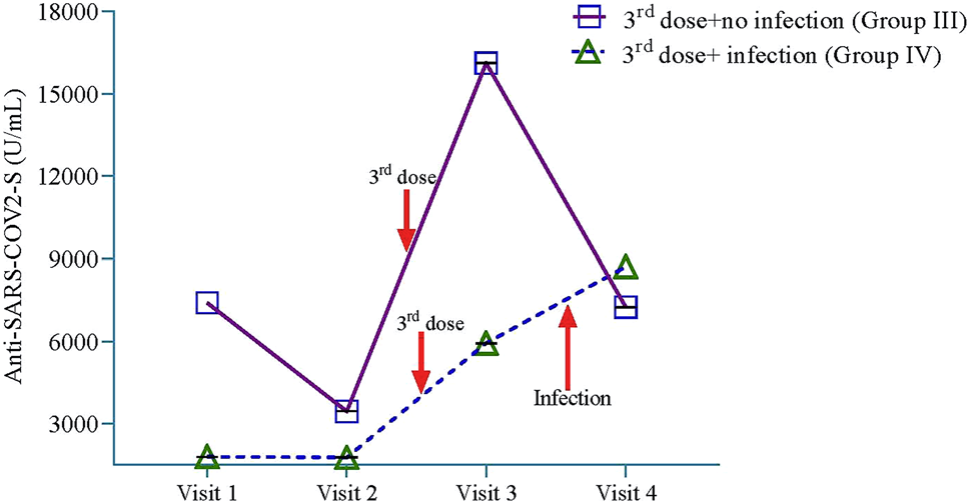
Kinetics of spike (S)-antibody titers in participants from Groups III and IV at visits 1, 2, 3 and 4. Data are presented as GM ± SD. Multivariate regression model was used to calculate the estimated mean and the regression model was adjusted by age, sex, household income, occupation, and BMI. Group III consists of individuals, who received the 3rd dose of vaccine between visit 2 and 3, and remained uninfected with SARS-CoV-2 (tested negative by RT-PCR, did not exhibit COVID-like symptoms, negative for N-antibodies) up to visit 4 (n = 55); Group IV participants received the 3rd dose of vaccine between visits 2 and 3, but got infected with SARS-CoV-2 (tested positive by RT-PCR or showed COVID-like symptoms and positive for N-antibodies) between visits 3 and 4 (n = 12). BMI: Body-mass index; GM: Geometric Mean; SD: Standard deviation; SARS-CoV-2: Severe Acute respiratory Coronavirus-2.

In group III participants (receiving the 3rd dose between visit 2 and 3, and remained uninfected in subsequent follow-ups), significant increase in S- antibody titers were noted at visit 3 after the receipt of the 3rd dose. Antibody titers declined significantly in the subsequent visit (visit 4), returning to the levels seen at enrollment (Fig. 2). Following the administration of the 3rd vaccine dose between visits 2 and 3 in Group IV participants (receiving the 3rd dose between visit 2 and 3, but got infected with SARS-CoV-2 between visits 3 and 4), the S-antibody titers at visit 3 was much lower compared to group III (5929 ± 4.24 vs. 16,107 ± 8.18); after infection there was an increment of the titers at visit 4 (Fig. 2). AstraZeneca vaccine group received the 3rd dose between visits 3 and 4, which led to increase in S- antibody titers at visit 4; however, the participants could not be followed beyond visit 4 (end line of the study).

### Protection from (re)infection with SARS-CoV-2

Applying Markov Chain Monte Carlo (MCMC) method, we found that the odds of protection from (re)infection with SARS-CoV-2 following primary vaccination in the first 5 months was higher in the mRNA and viral vector-based vaccine groups compared to inactivated vaccine group. Protection from (re)infection persisted in the subsequent 4 months in the viral vector-based vaccine group only. After 12 months the level of protection became insignificant (Table 4).

**Table 4:** Odds of protection from (re)infection in Pfizer, Moderna and AstraZecena vaccine recipients compared to Sinopharm vaccine recipients.

	Between visit 1 & 2			Between visit 2 & 3			Between visit 3 & 4		
	OR(95% CI)	*P* value	*MCSE	OR(95% CI)	*P* value	*MCSE	OR(95% CI)	*P* value	*MCSE
Sinopharm	Refs.			Refs.			Refs.		
Pfizer	0.08(0.04 0.14)	< 0.001	0.002	0.53(0.33, 1.08)	0.080	0.02	0.85(0.40, 1.81)	0.685	0.02
Moderna	0.15(0.11, 0.21)	0.001	0.004	0.52(0.35, 1.35)	0.212	0.01	0.51(0.22, 1.17)	0.112	0.004
AstraZeneca	0.13(0.09, 0.17)	0.001	0.002	0.06(0.05, 0.07)	< 0.001	0.0007	–	–	–

^#^Bayesian framework and MCMC methods were used to compare the effectiveness of different COVID-19 vaccines in reference to Sinopharm vaccine. The model was adjusted with age, sex, household income, occupation and BMI. ^#^Analysis included both group I [individuals who remained uninfected with SARS-CoV-2 (tested negative by RT-PCR, did not exhibit COVID-like symptoms, negative for N-antibodies) and did not receive a 3rd dose of vaccine during the one-year study period] and group II [individuals who got infected with SARS-CoV-2 (tested positive by RT-PCR or showed COVID-like symptoms and positive for N-antibodies) and did not receive a 3rd dose during the study period]. *a smaller MCSE indicates more precise parameter estimates, while a larger MCSE suggests greater uncertainty. *OR*: Odds ratio; *CI*: Confidence interval; *COVID-19*: Corona virus diseases 2019; *SARS-CoV-2*: Severe acute respiratory coronavirus-2; *RT-PCR*: Reverse transcription polymerase chain reaction; *MCMC*: Markov chain Monte Carlo; *MCSE*: Monte Carlo standard error.

We also evaluated the effect of pre-enrolment infection between 1st and 2nd dose of primary vaccination, on vaccine-mediated protection from re-infection with SARS-CoV-2 during the study period. Among the 70 participants who got infected before enrolment, 52 participants remained uninfected at visit 2. Excluding 3 drop-out participants, the uninfected participants account for 78%. Interestingly, the GM antibody titer of these uninfected participants (8650 ± 3.51) at visit 1 was significantly higher than that of infected participants (1357 ± 2.08) (*p* < 0.001). Excluding drop-outs and 3rd dose recipients from the 52 uninfected participants, 72% (26 out of 36) participants did not get infected between visits 2 and 3. Again, there was a significant difference in the S- antibody titers between uninfected (6166 ± 3.46) and infected participants at visit 2 (2692 ± 2.63) (*p* < 0.001). Analysis using MCMC method showed that pre-enrolment infection between 1st and 2nd doses of primary vaccination imparted 60% (95% confidence intervals (CI), 27%, 73%; MCSE = 0.017) protection against reinfection between visit 1 & 2 and 50% (95% CI, 43%, 60%; MCSE = 0.004) protection between visit 2 & 3). Between visits 3 and 4, 95% (20 out of 21) participants remained uninfected. Since only one participant got infected during this period, the antibody titers at visit 3 were not compared between uninfected and infected participants.

## Discussion

The present study describes the longevity and waning of anti-spike antibody titers in community participants in Bangladesh after receiving any of the COVID-19 vaccines available during 2021 to 2022 and the risk of (re)infection. S- antibody titers were reduced significantly at 5 months post vaccination, and continued to decline at subsequent visits in participants who remained uninfected with SARS-CoV-2 and did not receive the 3rd (booster) dose of vaccine. The 3rd dose of vaccination or an infection between any two consecutive visits after the primary vaccination led to increase in S-antibody titers in later visits. The odds of protection from (re)infection with SARS-CoV-2 following primary vaccination was higher in mRNA and viral vector-based vaccine groups compared to inactivated vaccine in the first 5 months, however, the likelihood of protection persisted in the viral vector-based vaccine group for a longer period, i.e. up to 9 months.

Irrespective of the vaccine type, the augmented S- antibody titers at enrollment i.e. at 2–4 weeks after the 2nd dose of COVID-19 vaccines, were reduced by about 50% at 4 months follow-up and continued to decline afterwards in individuals who remained uninfected with SARS-CoV-2. Consistent with this finding, previous studies have reported that S-antibody levels increase at 2–6 weeks post-vaccination with Pfizer and AstraZeneca vaccines, and the levels start declining from as early as 3 to 24 weeks^9,11,12^. Many studies have shown that peak antibody responses elicited by mRNA vaccines rapidly decline within 6–8 months post-vaccination^13–15^. However, there are also reports of longer durability (> 6 months) of SARS-CoV-2 specific IgG antibodies among health care workers after vaccination with mRNA vaccines^16,17^. In a multistate, longitudinal cohort study of around 13,000 US adults receiving mRNA vaccines, S-antibodies persisted up to around 9 months with only 2.4% of the study population exhibiting seroreversion^18^. The inactivated whole virus vaccines have been shown to produce a lower initial antibody response than mRNA vaccines and the levels fell below the positivity cut-off value (seroreversion) by 4 months after vaccination^19,20^. Likewise, our study also showed lower magnitude of antibodies generated by Sinopharm vaccine compared to other types of vaccines.

Persistence of elevated levels of anti-spike antibodies to SARS-CoV-2 in a population generated by vaccination or infection reflect protection from (re)infection with SARS-CoV-2^18,21,22^. Administrating vaccine to individuals with prior SARS-CoV-2 infection was shown to generate high level of durable binding and neutralizing antibody responses compared to uninfected individuals^23–25^. Concordantly, we found that SARS-CoV-2 infection between primary vaccination doses substantially increased post-vaccination antibody levels and bestowed vaccine-mediated protection from reinfection. Intriguingly, participants who became infected after vaccination (between any two visits) showed lower S-antibody titers at preceding visits compared to those who remained uninfected. Furthermore, 3rd dose of COVID-vaccine boosted the S-antibody titers and provided further protection against (re)infections (82% remain uninfected) for another 5–6 months. These finding reinforces the view that protection from further infection is largely linked to elevated levels of S-antibodies and waning antibody response predicts reduced protection from SARS-CoV-2 infection. On the contrary, some individuals did not get infected during the one-year study period even when their antibody levels declined by 82% of the levels found at visit 1 (2–4 weeks after primary dose). It is possible that other arms of the immune system namely cellular immunity and innate immunity play a crucial role in protection against infection^26,27^.

Administration of two primary doses of mRNA vaccine resulted in highest S-antibody titers followed by AstraZeneca and Sinopharm vaccines at 2–4 weeks after vaccination, as shown earlier by our group and others^28–32^. In terms of protection from (re)infection, the highest breakthrough infection post-vaccination was seen in the inactivated vaccine group (40%) as opposed to vector-based (16%) and mRNA vaccine groups (21% to 23%), indicating better protection by mRNA and vector-based vaccines compared to inactivated vaccine. These findings further underpin the protective role of antibodies. Additionally, we found that protection from (re)infection persisted only for 5–6 months after primary immunization in mRNA vaccine groups, while for vector-based vaccine group it persisted for a longer period. This result is somewhat different from the finding of two earlier studies. The ZOE COVID study showed that mRNA vaccines (Pfizer and Moderna) offer better effectiveness than the Oxford-AstraZaneca COVID-19 vaccine in UK population; however, both vaccine types showed a decline in effectiveness over 5 months^7^. In another study, increased risk of severe COVID-19 outcome was observed about 3 months after a second shot of both Pfizer and AstraZeneca vaccines^8^*.* Slightly lower breakthrough infection rate and longer duration of protection in AstraZeneca vaccine group might be partially explained by the role of cellular immunity, as shown in our earlier study^30^.

The study has a number of limitations. While our findings of the longitudinal follow-up provide valuable insights into the durability of vaccine specific antibodies and protection, we recognize that the small sample size diminished the robustness, and restricted the generalizability of the findings. Moreover, targeted number of participants could not be recruited as the roll-out of different COVID-19 vaccines was paused at different time points in Bangladesh. Studies with larger population of different age groups, while stratifying for sex would allow for a greater confidence in the observed findings. Another important limitation is the unavailability of PCR-based testing of all participants who reported symptoms. To overcome this limitation, we identified subclinical infections (reported as mild or moderate COVID-like symptoms by the volunteers) by testing for presence of anti-nucleocapsid antibodies, which are elicited by natural infection but not by the mRNA or the vector-based vaccine. Since N-protein is included in the Sinopharm vaccine, N-antibody response for confirmation of infection was not possible in the inactivated vaccine group. However, a boosting of S-antibody response indicated a subclinical infection in this vaccine group. The longevity of S-specific antibodies after the 3rd dose could not be followed beyond visit 4 in the AstraZeneca group, since this group received the 3rd dose between visits 3 and 4. Absence of unvaccinated group for comparing with vaccinated groups was another limitation, which was almost impossible to address, since > 60% of Bangladeshi population were vaccinated with available COVID-19 vaccines within six months after initiation of the study^33^. Another weakness was that virus neutralizing antibodies were not determined to demonstrate protection, although S-antibody titers have been shown to closely represent neutralizing antibodies^34,35^ Moreover, we did not study cellular and innate immune responses in the vaccinated and infected participants to better understand the protection afforded by the different arms of immunity.

In conclusion, our findings demonstrated that spike-specific antibodies decline after 5–6 months of administration of a COVID-19 vaccine dose, and the level of protection from (re)infections with SARS-CoV-2 appears to be linked to some extent with the antibody levels. The viral vector-based COVID vaccine apparently showed longest protection from future infection compared to mRNA or inactivated vaccines. Thus, vector-based vaccines may be recommended for elderly and people with comorbidities which offers longer protection, easier to transport and store, and is cheaper. Further prospective studies are needed where both humoral and cellular functional immune responses will be studied in parallel, to better understand the protective immunity in vaccinated individuals. More research using different vaccine types on vaccine-induced protection over extended period are warranted to help formulate effective COVID-19 vaccination and booster strategies.

## Methods

### Study design, setting and study population

Participants of this cohort study were recruited from Sheikh Russel Gastroliver Institute & Hospital (SRGIH), a public sector health facility in Dhaka, Bangladesh when they visited the hospital to receive second dose of COVID-19 vaccines. Adults who received two doses of COVID-19 vaccines (Oxford–AstraZeneca COVID‑19 vaccine or Covishield (viral vector-based vaccine), Pfizer-BioNTech (BNT162b2) (mRNA vaccine), Moderna (mRNA-1273) (mRNA vaccine) or Sinopharm (BBIBP-CorV) (inactivated whole virus vaccine)), were enrolled within 2–4 weeks of receiving the second primary dose (visit 1) and followed-up at 4 months (visit 2), 8 months (visit 3) and 12 months (visit 4). The inclusion criteria for participation were: (1) male or female adults (aged 18 years and above), (2) able to understand and sign the informed consent form, (3) available and reachable by study staff for the entire period of the study; (4) agreeing to provide blood sample for the research study. The exclusion criteria were: (1) residence outside of Dhaka city; (2) suffering from long term severe illness such as cancer, chronic obstructive pulmonary disease (COPD) and chronic kidney disease (CKD).

Enrolment of participant started in September 2021 and ended in July 2022. Bangladesh received different types of COVID-19 vaccines at different time points either through COVAX or direct procurement, therefore availability of vaccinated individuals with any particular type of COVID-19 vaccine varied by time. Therefore, enrolment of vaccine groups varied by months. The Covishield was the first COVID-19 vaccine that was rolled out in Bangladesh and thus our first enrolled vaccine group was AstraZeneca vaccine. Administration of booster dose started in December 2021 in limited hospitals among older peoples (> 60 years of age) and age limit was gradually descended towards younger population. The participants of the AstraZaneca vaccine group received booster dose after almost 8–9 months.

### Data and specimen collection

A structured questionnaire was utilized to collect data from each study participant at enrollment (visit 1, 2–4 weeks after administration of 2nd primary dose) and each follow-up visit, i.e. at 4 months (visit 2), 8 months (visit 3) and 12 months (visit 4) post second dose (Fig. 1). Collected data included socio-demographic information (age, sex, education, migration background, ethnicity, marital status, household structure, occupation, and income), influenza-or COVID-like symptoms or presence of confirmed COVID-19 cases currently and in the past 6-months; co-morbidities (e.g. diabetes, hypertension, stroke, heart diseases and asthma). The vaccination type and administration dates were recorded. Information on receipt of booster dose was also collected during the follow-up phase. Height and weight were collected using stadiometer (Seca 217, Hamburg, Germany) and digital weighing scale (Camry-EB9063, China), respectively. Venous blood was collected at each visit in Lithium-heparin coated tubes (S-Monovette Plasma, Sarstedt AG & Co. KG, Nümbrecht, Germany), plasma was separated upon centrifugation, aliquoted, and stored at -80 °C until use.

### Detection of SARS-CoV-2 spike protein (S)- and nucleocapsid (N)- specific antibodies

The concentration of S-specific antibodies was determined in plasma by Elecsys® Anti-SARS-CoV-2 S immunoassay kit (Roche Diagnostics GmbH, Mannheim). This assay allowed quantitative determination of high affinity antibodies, predominantly IgG, but also IgA and IgM directed to the SARS-CoV-2 spike (S) protein receptor binding domain (RBD) in a double-antigen sandwich assay format on Cobas-e601 analyzer (Roche Diagnostics). According to the kit insert, the sensitivity of the Elecsys Anti‑SARS‑CoV‑2 S assay is 98.8%, clinical specificity is 99.98%, and the assay was found to have 92.3% positive agreement rate with a Vesicular Stomatitis Virus (VSV)-based pseudo-neutralization assay.

Nucleocapsid (N)-specific antibodies was determined in plasma by Elecsys® Anti-SARS-CoV-2 immunoassay kit (Roche Diagnostics). The kit allows simultaneous detection of mature Nucleocapsid-specific IgM and IgG antibodies on an automated immunoassay analyzer (Cobas-e601, Roche Diagnostics). This is a qualitative assay that gives combined antibody titers of both IgM and IgG and does not differentiate between the two types. Based on the antibody cut-off index (COI), the serological response to SARS-CoV-2 is categorized as reactive (COI ≥ 1.0, seropositive) and non-reactive (COI < 1.0, seronegative). According to the kit insert, the Elecsys Anti-SARS-CoV-2 assay has 99.8% specificity and > 99.5% sensitivity.

All participants vaccinated with viral vector-based vaccines (AstraZeneca) and mRNA vaccines (Pfizer and Moderna) were tested for N-antibodies to identify previous exposure to or infection with SARS-CoV-2. Participants immunized with Sinopharm vaccine (inactivated whole virus) were not tested for N antibodies as it is not possible to differentiate the N-antibodies induced by vaccination from those generated due to natural infection with SARS-CoV-2.

### Infection status

The study participants were defined as SARS-CoV-2 infected when tested positive by RT-PCR or showed COVID-like symptoms and concomitantly positive for N-antibodies. Individuals who did not exhibit COVID-like symptoms and were negative for N- antibodies or tested negative by RT-PCR were considered uninfected with SARS-CoV-2.

### Ethics approval

The authors affirm that all procedures involved in this research adhered to the ethical standards set by the pertinent national and institutional committees overseeing human experimentation, aligning with the Helsinki Declaration of 1975, revised in 2008. The study received approval from the institutional review board of icddr,b (PR-21069, dated 17 August 2021). Written informed consent was obtained from the participants.

### Statistical analysis

The sample size was calculated based on the primary endpoint, i.e. to assess the persistence of SARS-CoV-2 S-specific antibody response following two primary doses of COVID-19 vaccines. A study by Shrotri M et al*.*^12^, demonstrated a decline of antibody titers by 19.7% from 0 to 21 days to 22–41 days after administration of two doses of Pfizer vaccine. Based on this information, and considering statistical power of 80% and confidence interval of 95%, the estimated sample size was 64 per vaccine group. To reduce unknown bias due to different localities and socio demographic status, and unknown effects of COVID-19 vaccination, a design effect of 2 was added, which resulted in a sample size of 128. Considering 10% attrition rate, the final sample size was 140.8 (rounded to 141) per vaccine group and the total sample was 564. Despite rigorous efforts, we encountered difficulties in recruiting the target number of participants in the study. Within the duration of the study, administration of different COVID-19 vaccines was paused at different period. We were able to enroll only 452 participants, with the highest number in AstraZaneca vaccine group.

We showed basic demographic characteristics of the study participants, categorized by the respective vaccine types received (Pfizer, Moderna, AstraZeneca, and Sinopharm).

S-antibody data exhibited a right-skewed distribution in a histogram. To address this non-normal distribution, a natural log transformation was employed. To analyze the durability of S-antibody titers at different visits post-primary vaccination (primary endpoint), the participants across all vaccine groups were categorized into two groups: Group I consisted of individuals who remained uninfected with SARS-CoV-2 (defined above) and did not receive a 3rd dose of vaccine during the one-year study period. Group II included participants who got infected with SARS-CoV-2 (defined above), and did not receive a 3rd dose of vaccine during the study period. Furthermore, to assess the kinetics of S- antibody titers after the 3rd dose (secondary endpoint 1), the recipients were divided into 2 groups: Group III consisted of individuals who received the 3rd dose of vaccine between visit 2 and 3, and remained uninfected up to visit 4 (n = 55); Group IV participants received the 3rd dose of vaccine between visits 2 and 3, but got infected with SARS-CoV-2 between visits 3 and 4 (n = 12). Since the number of 3rd dose recipients was relatively small, the analysis in group III and IV was not stratified based on the type of COVID-19 vaccine received. Moreover, the AstraZeneca group was excluded from this analysis as this group received the 3rd dose between visit 3 and 4 (n = 105), and could not be followed further after completion of 1-year follow-up to observe the durability of S-antibodies. To evaluate the mean differences in S-antibody titers between any two visits in each of the groups (groups I to IV), a multivariate regression model was employed. Repeated measure ANCOVA model was employed to examine the mean differences in S-antibody titers between multiple visits within a group. In order to determine the optimal model, age, sex, household income, occupation, body mass index (BMI), education, comorbidities, etc. were incorporated as covariates in the regression model. The effects of education and comorbidities was found minimal (< 1%), thus, not included in the final regression model.

To assess the degree of protection from infection with SARS-CoV-2 (secondary endpoint 2) in mRNA and viral vector-based vaccine groups compared to inactivated vaccine as the reference group, Bayesian framework was adopted and Markov Chain Monte Carlo (MCMC) method was utilized to derive posterior distributions of model parameters. Furthermore, we applied model evaluation techniques and conducted sensitivity analyses to ensure the robustness of our results.

All statistical analyses were conducted using STATA (version 15) and Python (3.11), and GraphPad Prism was utilized for generating graphs. Significance was defined as a *p*-value of < 0.05.

### Supplementary Information

Supplementary Information.

## Data Availability

All data underlying the findings in our study are freely available in the manuscript and supplemental files. Additional data that support the findings will be available upon reasonable request to corresponding author as per icddr,b policy (http://www.icddrb.org/policies).
