# Author Correction: Antibody longevity and waning following COVID‑19 vaccination in a 1‑year longitudinal cohort in Bangladesh

**DOI:** 10.1038/s41598-024-66734-2

**Published:** 2024-07-08

**Authors:** Md. Ahsanul Haq, Anjan Kumar Roy, Razu Ahmed, Rakib Ullah Kuddusi, Monika Sinha, Md. Shamim Hossain, Maya Vandenent, Mohammad Zahirul Islam, Rashid U. Zaman, Md. Golam Kibria, Abdur Razzaque, Rubhana Raqib, Protim Sarker

**Affiliations:** 1 Immunobiology, Nutrition and Toxicology Laboratory, Nutrition Research Division, International Center for Diarrhoeal Disease Research, Bangladesh (icddr, b), Dhaka, 1212 Bangladesh; 2 UNICEF, Dhaka, 1207 Bangladesh; 3 Embassy of Sweden in Bangladesh, Dhaka, 1212 Bangladesh; 4 British High Commission, Dhaka, 1212 Bangladesh; 5 Sheikh Russel Gastroliver Institute and Hospital, Dhaka, 1212 Bangladesh

Correction to: *Scientific Reports* 10.1038/s41598-024-61922-6, published online 20 May 2024

The Funding section in the original version of this Article was omitted. The Funding section now reads:

“This work was funded by The Foreign, Commonwealth & Development Office (FCDO; GR-02142), The United Nations Children’s Fund (UNICEF; GR-02141) and The Institute of Development Studies (IDS; GR-02144). Additionally, icddr, b extends its thanks to the Governments of Bangladesh and Canada for their core/unrestricted support.”

The original article has been corrected.

