# Supplementary material for: Antibody longevity and waning following COVID-19 vaccination in a 1-year longitudinal cohort in Bangladesh
Source: Sci Rep. 2024 May 20;14:11467. doi: 10.1038/s41598-024-61922-6 (PMC11106241; doi:10.1038/s41598-024-61922-6)
Supplement: Supplementary file 1 — Supplementary Information. [file 41598_2024_61922_MOESM1_ESM.docx]

**Antibody longevity and waning following COVID-19 vaccination in a**

**one-year longitudinal cohort in Bangladesh**

Md. Ahsanul Haq^1 §^, Anjan Kumar Roy^1 §^, Razu Ahmed^1^, Rakib Ullah Kuddusi^1^, Monika Sinha^1^, Md. Shamim Hossain^1^, Maya Vandenent^2^, Mohammad Zahirul Islam^3^, Rashid U. Zaman^4^, Md. Golam Kibria^5^, Abdur Razzaque^1^, Rubhana Raqib^1^, Protim Sarker*^1^

^1^International Center for Diarrhoeal Disease Research, Bangladesh (icddr,b), Dhaka-1212, Bangladesh; ^2^UNICEF, Dhaka-1207, Bangladesh; ^3^Embassy of Sweden in Bangladesh, Dhaka-1212, Bangladesh; ^4^British High Commission, Dhaka-1212, Bangladesh; ^5^Sheikh Russel Gastroliver Institute & Hospital, Dhaka 1212, Bangladesh

^§^equal contribution as first authors

^*^Corresponding author:

Protim Sarker, Ph.D.

Immunobiology, Nutrition and Toxicology Laboratory

Nutrition Research Division

Icddr,b

Email: [protim@icddrb.org](mailto:protim@icddrb.org)

**Supplementary Figure 1.** A scatter plot showing decrease in antibody titers over time in participants (n=20) who were infected with SARS-CoV-2 during primary dosing with COVID-19 vaccines (between 1^st^ and 2^nd^ doses), completed all four follow-up visits, did not get re-infected and did not receive a third dose. Repeated measure ANCOVA model was applied to estimate the decline in antibody titers and p-values, and the model was adjusted by age, sex, household income, occupation, and BMI. The data are shown as mean ± SD. BMI: Body-mass index; COVID-19: Corona virus diseases 2019; SARS-CoV-2: Severe Acute respiratory Coronavirus-2; SD: Standard deviation.

|  |
| --- |
|  |

**Supplementary Figure 2**. A bar chart depicting difference in S-IgG antibody titers between infected and uninfected participants at preceding and following visits of infection with SARS-CoV-2; (A) Infection between visits 1 and 2, (B) Infection between visits 2 and 3. Data are given as GM ± SD. Multivariate regression model was used to estimate the p-value and the regression model was adjusted by age, sex, household income, occupation, and BMI. S BMI: Body-mass index; GM: Geometric mean; SD: Standard deviation; SARS-CoV-2: Severe Acute respiratory Coronavirus-2.
